# The No-Touch Saphenous Vein Harvesting Improves Graft Patency After Off-Pump Coronary Artery Bypass Surgery: A Propensity-Matched Analysis

**DOI:** 10.21470/1678-9741-2022-0189

**Published:** 2022

**Authors:** Zhan Peng, Rui Zhao, Zhiguang Liu, Yuhua Liu, Yunxiao Yang, Xiubin Yang, Kun Hua

**Affiliations:** 1 Department of Cardiovascular Surgery, Beijing Anzhen Hospital, Capital Medical University, Beijing Institute of Heart, Lung and Vessel Disease, Beijing, People’s Republic of China; 2 Fuwai Hospital, National Center for Cardiovascular Diseases, Chinese Academy of Medical Sciences and Peking Union Medical College, Beijing, People’s Republic of China

**Keywords:** Saphenous Vein, Off-Pump, Coronary Artery Bypass, Propensity Score, Wound Injuries, Hypesthesia.

## Abstract

**Introduction:**

This single-center study of propensity-matched data was performed to assess the effect of the no-touch saphenous vein (NTSV) harvesting technique on early- and long-term outcomes of patients after off-pump coronary artery bypass grafting (OPCABG) in China.

**Methods:**

A retrospective analysis of 767 patients who underwent OPCABG in the Beijing Anzhen Hospital (June 2017 to October 2021) was performed, and their data entered the conventional saphenous vein (CSV) harvesting technique group or the NTSV group. In-hospital and follow-up outcomes were evaluated by adjusting baseline characteristics using propensity score matching (1:1). Clinical outcomes and postoperative angiographic results were compared.

**Results:**

The saphenous vein graft patency rates at postoperative three months and one year for the NTSV group vs. CSV group were 99.6% vs. 96.2% (P<0.001) and 97.3% vs. 93.1% (P<0.001), respectively. The two matched groups received a significantly different cumulative incidence function of saphenous vein graft occlusion for the longer follow-up period in Kaplan-Meier curves (χ^^2^^=4.330, log-rank P=0.037). No difference in early- and long-term mortality or major adverse cardiac and cerebrovascular events (MACCE) were observed between the groups. The rate of MACCE was not statistically significant different between the groups, but there was a tendency favoring the no-touch technique (9.8% CSV vs. 4.8% NTSV; P=0.067). More patients in the NTSV group developed postoperative leg wound exudation (5.4% vs. 1.2%; P=0.032) and skin numbness (22.2% vs. 8.9%; P=0.001) than in the CSV group.

**Conclusion:**

The NTSV is an excellent conduit to be used in OPCABG. There remains a need to reduce leg wound complications.

**Table:** 

Abbreviations, Acronyms & Symbols			
BMI	= Body mass index	MACCE	= Major adverse cardiac and cerebrovascular events
CABG	= Coronary artery bypass grafting	MI	= Myocardial infarction
CHF	= Chronic heart failure	NTSV	= No-touch saphenous vein
COPD	= Chronic obstructive pulmonary disease	NYHA	= New York Heart Association
CSV	= Conventional saphenous vein	OPCABG	= Off-pump coronary artery bypass grafting
CT	= Computed tomography	PCI	= Percutaneous coronary intervention
IABP	= Intra-aortic balloon pump	PDA	= Posterior descending artery
ICU	= Intensive care unit	PS	= Propensity score
ITA	= Internal thoracic artery	RCA	= Right coronary artery
LAD	= Left anterior descending coronary artery	RIMA	= Right internal mammary artery
LCX	= Left circumflex coronary artery	SD	= Standard deviation
LIMA	= Left internal mammary artery	SV	= Saphenous vein
LVEDD	= Left ventricular end-diastolic diameter	SVG	= Saphenous vein graft
LVEF	= Left ventricular ejection fraction	TNI	= Troponin I

## INTRODUCTION

Ischemic heart disease, the leading cause of death worldwide, is expected to account for 14.2% of all deaths by 2030^[^1^]^. Coronary artery bypass grafting (CABG) is one of the most common interventions globally for complicated and advanced coronary heart disease^[^2^]^. Graft patency is an important determinant of long-term clinical success after CABG^[^3^]^. The most universal used conduit continues to be the saphenous vein graft (SVG). Nonetheless, vein grafts are subject to a relatively high occurrence rate of occlusion compared with arterial grafts. The current studies show that the occlusion of SVG reached up to 13% at one month and 30% within one year after CABG^[^4^-^7^]^, which is associated with adverse cardiovascular events. Among numerous efforts to overcome the structural and functional limitations of SVG, it was introduced the no-touch saphenous vein (NTSV) harvesting technique, where the saphenous vein (SV) is harvested with a pedicle of surrounding tissue^[^5^]^. Previous remarkable studies reported significantly less occlusion using NTSV, which was 5.5% at 1.5 year^[^9^]^ to 10% at 8.5 years^[^10^]^. These studies were primarily conducted in high-income countries, but it remains unknown whether this marked effectiveness could be generalized in China, where CABG volume is one of the highest in the world and where the vein graft is dominant in CABG. The present study was performed to assess the clinical and angiographic outcomes in patients who received NTSV grafts after off-pump coronary artery bypass grafting (OPCABG) in the Beijing Anzhen Hospital.

## METHODS

### Study Design

**Fig. 1:**
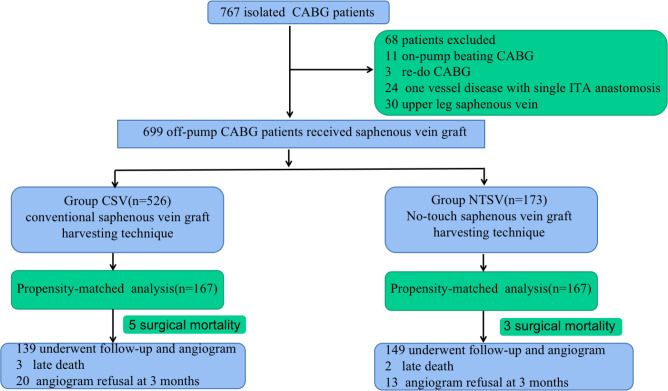
Summary flow diagram of enrolled patients. CABG=coronary artery bypass grafting; CSV=conventional saphenous vein; ITA=internal thoracic artery; NTSV=no-touch saphenous vein.

This study protocol was approved by the ethics committee of Beijing Anzhen Hospital and was consistent with the Declaration of Helsinki. From June 2017 to October 2021, 767 consecutive patients underwent isolated OPCABG in our center. Among those, 68 patients (8.8%) who underwent redo CABG, on-pump beating CABG, one vessel disease with single internal thoracic artery anastomosis, or received upper leg SV were excluded (Figure 1). Therefore, 699 patients were included in the present study with the SV harvested from the lower leg. All included patients were divided into the conventional saphenous vein (CSV) harvesting technique group (n=526) or the NTSV group (n=173). Perioperative clinical and baseline data were collected from the institutional database system, and follow-up data were obtained using standardized forms during telephone or clinic visits. The propensity score (PS) matching model was performed to adjust baseline differences in consideration of potential confounding factors and the effects of treatment selection bias. In-hospital outcomes included surgical mortality (death in 30 days or the same hospitalization after operation) and in-hospital morbidity (respiratory complications, infection, re-exploration for bleeding, stroke, renal dysfunction, myocardial infarction associated with CABG). Respiratory complications included prolonged ventilator support > 48 hours or pneumonia after surgery. Renal dysfunction was defined as the serum creatinine level increasing > 50% or the need of continuous renal replacement therapy. The occurrence of postoperative atrial fibrillation was defined as any short runs of atrial fibrillation > 30 seconds.

**Supplement Fig. 1:**
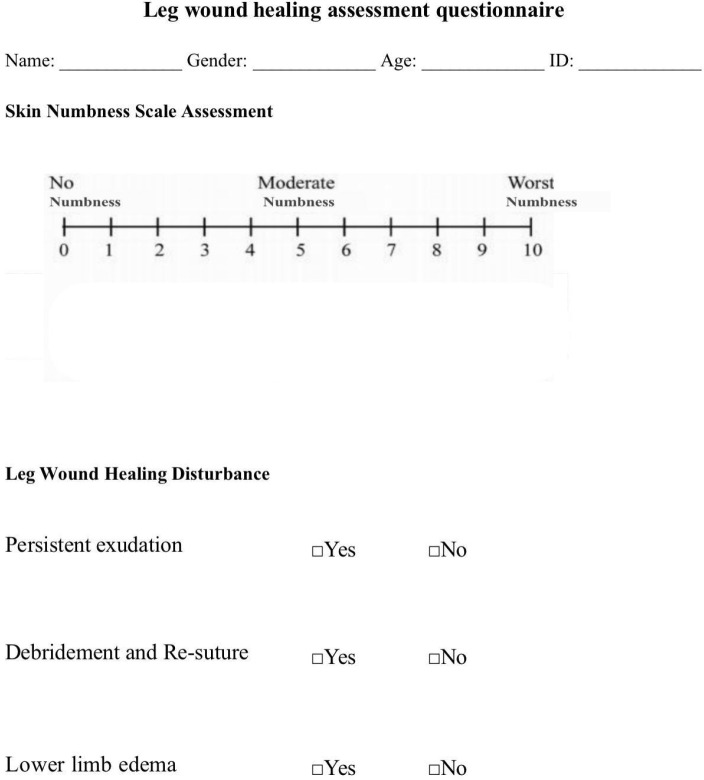
The skin numbness scale assessment questionnaire. ID=identity.

Follow-up outcomes included graft patency, all-cause mortality, major adverse cardiac and cerebrovascular events (MACCE), and leg wound complication. Graft patency was evaluated by multislice computed tomography (CT) angiography or coronary angiography. Angiographic outcomes were reviewed by physician (coronary angiography), radiologist (multidetector CT angiography), and the author of this study (all of the angiographies) to reach consensus; all of the angiography reviewers were blinded to the SV harvesting techniques of patients. Graft occlusion was defined as the graft conduit not filled with contrast but with a string sign found in any segment and any occlusion of the distal anastomoses for sequential anastomosis depending on the FitzGibbon criteria^[^10^]^. For sequential anastomosis, one occlusion of any of the distal anastomoses was considered as occlusion of the whole graft vessel. MACCEs included cardiac-cause mortality, myocardial infarction, repeat revascularization, and cerebrovascular accident. Leg wound complications included wound infection, skin numbness, edema, persistent exudation, and any complication that needed re-suture. Skin numbness was measured by visual analogue scale (Supplement Figure 1), with scores ≥ 5 indicating skin numbness. Edema was defined as the tissues around leg incisions to swell after surgery, and persistent exudation was defined as continuous leak of blood components and interstitial fluid from lower limb incisions to prevent wound healing.

### Surgical Techniques and Postoperative Care

**Fig. 2:**
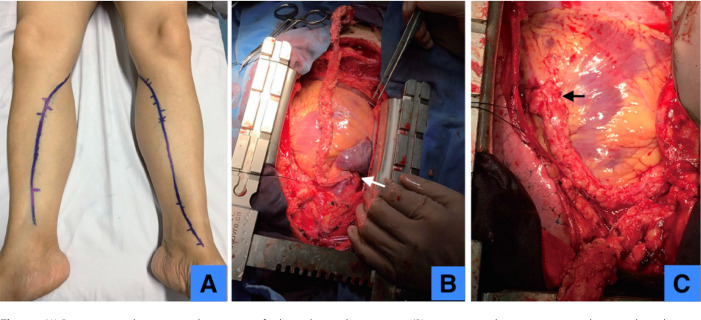
(A) Preoperative ultrasonography mapping for lower leg saphenous vein; (B) anastomosis between aorta and no-touch saphenous vein; (C) anastomosis between no-touch saphenous vein and target coronary artery.

In our center, an SV harvesting was performed by a senior surgeon who had previously traveled to Sweden to learn operative details from Professor Souza’s team. Doppler ultrasonography mapping was performed to assess the vein branches and quality of the SV to reduce damage before surgery (Figure 2). After anesthetic induction, longitudinal incisions on lower legs were performed using an open technique. The SV’s pedicle was harvested, with systemic heparinization for activated clotting time > 300 seconds, along with an approximately 5-mm wide margin of adjacent adipose tissues on both sides of the SV and thin layers of adherent connective tissues by the electrocautery knife with lower energy (20-30 J). Forced manual distension of the SV’s pedicle was not permitted. For conventional technique, the vein was stripped off its adventitia by blunt dissection with scissors, and all visible side branches were ligated or clipped by using an open technique, then the vein was removed and gently distended by heparinized saline. Leg incisions were carefully sutured by two layers of continuous suture. OPCABG was performed through a median full sternotomy. The internal mammary artery, SV, and radial artery were sequentially or separately grafted in the target coronary arteries. The transit-time flow probe (Medistim Butterfly Flowmeter, Oslo, Norway) was used to assess the quality of anastomosis, and reanastomosis would be considered when measured pulsatility index stood < 5. Subcutaneous injection of low molecular weight heparin within six hours, statin, aspirin, nitrates, and clopidogrel were routinely given to all patients after surgery, and clopidogrel was discontinued after one year. The application of other concomitant medications was depended on the patient’s condition.

### Statistical Analysis

Baseline characteristics are represented as the means with standard deviations for continuous variables, and these variables were compared by using the Student’s *t*-test. The Chi-squared test or Fisher’s exact test was performed for categorical variables. The Mann-Whitney-U test was used to compare non-normally distributed continuous data, which were represented as medians with interquartile ranges, and the Wilcoxon rank-sum test was performed as appropriate. PS matching was performed, and the non-parsimonious logistic regression propensity model included the following 13 variables: age; sex; obesity (body mass index > 30 kg/m^2^); smoking history; New York Heart Association (or NYHA) class III-IV; chronic obstructive pulmonary disease; history of stroke; diabetes mellitus; renal dysfunction; hypertension; history of percutaneous coronary intervention; left main artery disease; and emergency operation. PSs were created to quantify the likelihood of a given patient receiving NTSV harvesting technique. Using a 1:1 ratio matched pair design, we matched the NTSV group and CSV group subjects on the logit of the PS using calipers of width equal to 0.2 of the standard deviation of the logit of the PS. A greedy (nearest-neighbor) matching algorithm was used to form the pairs. After PS matching, the covariate balance was assessed using standardized mean differences, with values < 0.2 reflecting adequate balance. The McNemar and paired Student’s *t*-tests were used for comparisons with the matched groups. The generalized estimating equation for clustered binary outcome was used to analyze the graft patency rates. The Kaplan-Meier method and the log-rank test were used to compare the intervals to graft occlusion and MACCE, and we also performed the stratified log-rank test to reduce confounders. A value of two-sided *P*<0.05 was considered statistically significant, and data were analyzed using the IBM Corp. Released 2013, IBM SPSS Statistics for Windows, version 22.0, Armonk, NY: IBM Corp.

## RESULTS

Patient Characteristics and Perioperative Clinical Data

The baseline demographic and clinical data of the patients are listed in Table 1. Patients in the study cohort who were grouped into the NTSV group (n=173) compared with those grouped in the CSV group (n=526) presented no differences in characteristic and perioperative clinical data, except for a higher proportion of diabetics in CSV group than in NTSV group (*P*=0.011). PSs were then calculated, and the area under the receiver operating characteristic curve was 0.68 (95% confidence interval, 0.54-0.81; *P*=0.038); the Hosmer-Lemeshow goodness was 13.872 (*P*=0.673). There were 167 pairs of patients selected by PS matching (Table 1). Patients in the matched NTSV group compared with the matched CSV group were similar in characteristic and perioperative clinical data. After the PS matching, the difference in diabetics between the groups was no longer observed (18.3% [31 patients] NTSV *vs.* 20.1% [33 patients] CSV; *P*=0.781). The perioperative clinical data of the patients are listed in Table 2. Patients in the NTSV group compared with those in the CSV group had longer hospitalization time before surgery for the total cohort (9.3±4.6 days *vs.* 7.4±4.0 days; *P*=0.009) and for the propensity-matched cohort (8.1±4.2 days *vs.* 6.7±3.6 days; *P*=0.013), and longer duration of operation for the total cohort (4.4±0.6 hours *vs.* 3.2±0.8 hours; *P*=0.017) and for the propensity-matched cohort (4.2±0.3 hours *vs.* 3.1±0.5 hours; *P*<0.001). There were no statistically significant differences in graft type and the number of grafts between the two groups before and after PS matching.

**Table 1:** Preoperative characteristics of total cohort and propensity-matched cohort.

Characteristic	Total cohort (n=699)				Propensity-matched cohort (n=334)			
	CSV group (n=526)	NTSV group (n=173)	SD (%)	*P*-value	CSV group (n=167)	NTSV group (n=167)	SD (%)	*P*-value
**Preoperative clinical profile**								
Age (years)	61.3±7.2	62.7±8.2	8.3	0.678	60.6±8.5	61.2±8.6	7.2	0.773
Male [% (n)]	82.1% (431)	78.1% (135)	10.2	0.256	66.9% (111)	68.4% (114)	6.5	0.726
Height (cm)	168.0±6.4	165.6±6.7	9.6	0.351	166.0±5.4	162.0±7.2	7.1	0.618
BMI (Kg/m^2^)	24.9±4.4	24.0±4.0	13.7	0.195	24.3±3.4	23.9±3.1	8.4	0.538
Hypertension [% (n)]	25.8% (135)	33.6% (58)	15.2	0.045	34.8% (58)	33.0% (55)	4.2	0.729
Diabetes [% (n)]	30.4% (159)	20.1% (35)	22.3	0.011	20.1% (33)	18.3% (31)	4.1	0.781
Dyslipidemia [% (n)]	39.7% (208)	45.1% (78)	13.2	0.198	41.2% (69)	43.1% (73)	7.5	0.658
Recent smoking [% (n)]	31.3% (165)	29.4% (51)	7.4	0.641	29.3% (49)	28.4% (47)	3.6	0.809
COPD [% (n)]	3.8% (20)	4.2% (7)	2.6	0.885	4.1% (7)	3.7% (6)	2.8	0.777
History of PCI [% (n)]	16.1% (85)	14.0% (24)	9.2	0.472	12.5% (21)	11.3% (19)	2.5	0.736
History of stroke [% (n)]	13.2% (69)	13.4% (23)	1.2	0.952	14.6% (24)	11.9% (20)	7.9	0.518
History of CHF [% (n)]	9.2% (48)	8.8% (15)	2.1	0.856	4.3% (7)	2.9% (5)	7.3	0.557
Atrial fibrillation [% (n)]	8.5% (45)	6.7% (11)	8.7	0.356	6.5% (11)	6.5% (11)	< 0.1	1.000
NYHA III-IV	23.9% (125)	39.4% (68)	21.2	0.001	27.5% (46)	32.1% (54)	11.7	0.339
Emergency operation [% (n)]	5.3% (28)	4.5% (8)	6.3	0.718	4.1% (7)	3.7% (6)	3.7	0.777
Creatinine (umol/L)	99.2±85.2	91.4±32.2	8.2	0.414	89.2±75.2	85.2±25.2	4.7	0.618
TNI (ng/ml)	0.3±0.7	0.4±0.5	4.7	0.730	0.2±0.8	0.3±0.4	3.8	0.637
Carotid artery stenosis [% (n)]	24.7% (129)	25.9% (45)	7.1	0.695	26.9% (45)	26.9% (45)	< 0.1	1.000
**Angiographic and echocardiographic data**								
LVEF (%)	48±11	50±7	6.5	0.522	47±6	49±8	2.9	0.545
> 50% [% (n)]	76.0% (400)	60.5% (105)	23.4	0.001	61.7% (103)	62.3% (104)	1.8	0.910
30-50% [% (n)]	17.6% (93)	31.9% (55)	25.1	0.001	22.9% (38)	25.6% (43)	7.0	0.523
< 30% [% (n)]	6.3% (33)	7.5% (13)	7.2	0.568	4.5% (8)	6.4% (11)	8.7	0.579
LVEDD (mm)	49.1±5.6	47.5±5.3	3.8	0.711	47.1±3.6	46.5±2.3	3.7	0.646
Ventricular aneurysm [% (n)]	2.6% (14)	1.8% (3)	9.4	0.492	1.3% (2)	0.9% (1)	4.9	0.562
Left main disease [% (n)]	32.3% (169)	27.4% (47)	11.6	0.221	19.2% (32)	18.8% (31)	2.5	0.889
Number of coronary lesions	4 [3,5]	3 [1,4]	10.2	0.321	4 [3,5]	3 [1,4]	4.9	0.676
LAD [% (n)]	92.5% (487)	93.2% (161)	2.5	0.834	95.8% (160)	95.8% (160)	< 0.1	1.000
Diagonal [% (n)]	43.0% (226)	62.1% (107)	20.8	0.001	62.9% (105)	62.3% (104)	1.6	0.911
LCX [% (n)]	76.0% (399)	68.0% (118)	17.5	0.047	66.5% (111)	65.8% (110)	1.9	0.908
RCA [% (n)]	72.0% (378)	71.4% (123)	2.8	0.846	72.5% (121)	71.8% (120)	1.7	0.903
PDA [% (n)]	64.0% (337)	57.1% (99)	14.2	0.107	56.8% (95)	57.5% (96)	1.2	0.912

BMI=body mass index; CHF=chronic heart failure; COPD=chronic obstructive pulmonary disease; CSV=conventional saphenous vein; LAD=left anterior descending coronary artery; LCX=left circumflex coronary artery; LVEDD=left ventricular end-diastolic diameter; LVEF=left ventricular ejection fraction; NTSV=no-touch saphenous vein; NYHA=New York Heart Association; PCI=percutaneous coronary intervention; PDA=posterior descending artery; RCA=right coronary artery; SD=standard deviation; TNI=troponin I

**Table 2:** Perioperative variables for total and propensity-matched cohorts.

Variable	Total cohort (n=699)			Propensity-matched cohort (n=334)		
	CSV group (n=526)	NTSV group (n=173)	*P*-value	CSV group (n=167)	NTSV group (n=167)	*P*-value
Hospitalization time before surgery (days)	7.4±4.0	9.3±4.6	0.009	6.7±3.6	8.1±4.2	0.013
Hospitalization time after surgery (days)	10.7±6.3	10.8±6.8	0.216	10.1±4.2	10.3±5.4	0.327
Duration of operation (hours)	3.2±0.8	4.4±0.6	0.017	3.1±0.5	4.2±0.3	< .001
ICU staying (hours)	28±6.8	27.1±7.1	0.870	26.3±5.2	25.3±6.2	0.532
Time of mechanical ventilation (hours)	24±4.3	21.5±2.1	0.880	22±5.1	19.8±3.4	0.437
Number of grafts	4 [3,5]	3 [2,3]	0.325	4 [3,5]	3 [2,3]	0.561
IABP support [% (n)]	8.7% (45)	6.3% (11)	0.214	5.1% (9)	4.9% (8)	0.324
Graft type						
Vein [% (n)]	70.1% (1262)	70.0% (311)	0.004	69.5% (384)	64.7% (300)	0.096
*In situ* LIMA [% (n)]	26.4% (476)	27.3% (169)	0.001	28.8% (159)	34.5% (160)	0.052
Free LIMA [% (n)]	1.4% (25)	1.5% (7)	0.954	0.7% (4)	0.4% (2)	0.543
*In situ* RIMA [% (n)]	0.9% (16)	0.5% (2)	0.283	0.4% (2)	0.2% (1)	0.668
Radial [% (n)]	1.2% (22)	0.7% (3)	0.247	0.5% (3)	0.2% (1)	0.406

CSV=conventional saphenous vein; IABP=intra-aortic balloon pump; ICU=intensive care unit; LIMA=left internal mammary artery; NTSV=no-touch saphenous vein; RIMA=right internal mammary artery

### In-Hospital Mortality and Complications in the Propensity-Matched Cohort

Surgical mortality and major postoperative morbidity are shown in Table 3. There was no significant difference in surgical mortality between the two groups (1.8% NTSV *vs.* 2.7% CSV; *P*=0.474). Infection, renal insufficiency, re-exploration for bleeding, perioperative myocardial infarction, perioperative stroke, and prolonged ventilation were similar between the two groups. For leg wound complication, patients in the NTSV group developed a higher proportion of persistent exudation postoperatively (5.4% *vs.* 1.2%; *P*=0.032) and skin numbness (22.2% *vs.* 8.9%; *P*=0.001) than those in the CSV group. But there was no significant difference in re-suture before discharge between the two groups (2.9% NTSV *vs.* 1.6% CSV; *P*=0.474). No patient had severe wound complications such as necrosis or compartment syndrome.

**Table 3:** Clinical outcomes for propensity-matched cohorts.

Outcomes [% (n)]	CSV group (n=167)	NTSV group (n=167)	*P*-value
**Postoperative event**			
Surgical mortality	2.7% (5)	1.8% (3)	0.474
MI associated with CABG	2.7% (5)	1.8% (3)	0.474
Infection	1.8% (3)	0% (0)	0.082
Renal dysfunction	5.5% (9)	2.7% (5)	0.275
Re-exploration for bleeding	4.5% (8)	1.8% (3)	0.125
Respiratory complication	3.6% (6)	1.8% (2)	0.152
Stroke	6.4% (11)	2.7% (5)	0.124
Leg wound complication			
Persistent exudation	1.2% (2)	5.4% (9)	0.032
Skin numbness	8.9% (15)	22.2% (37)	0.001
Leg edema	19.8% (33)	20.3% (34)	0.891
Re-suture before discharge	1.6% (3)	2.9% (5)	0.474
Follow-up			
All-cause mortality	1.6% (3)	1.1% (2)	0.652
MACCE			
Cardiac-cause mortality	1.3% (2)	1.0% (1)	0.562
Repeat revascularization	3.8% (6)	1.8% (3)	0.311
MI	2.4% (4)	0.6% (1)	0.176
Stroke	2.4% (4)	1.8% (3)	0.702
**3-month graft patency**	(N=142)	(N=151)	
Saphenous vein patency (per graft)	96.2% (343/357)	99.6% (276/277)	< 0.001
ITA patency (per graft)	99.3% (139/140)	99.1% (149/151)	0.951
**12-month graft patency**	(N=139)	(N=149)	
Saphenous vein patency (per graft)	93.1% (295/317)	97.3% (230/237)	< 0.001
ITA patency (per graft)	98.3% (135/138)	97.6% (138/142)	0.423
Leg wound complication			
Persistent pain	7.4% (12)	8.4% (14)	0.683
Surgical intervention	4.2% (7)	4.6% (8)	0.792

CABG=coronary artery bypass grafting; CSV=conventional saphenous vein; ITA=internal thoracic artery; MACCE=major adverse cardiac and cerebrovascular events; MI=myocardial infarction; NTSV=no-touch saphenous vein

### Angiographic Outcomes in the Propensity-Matched Cohort

**Fig. 3:**
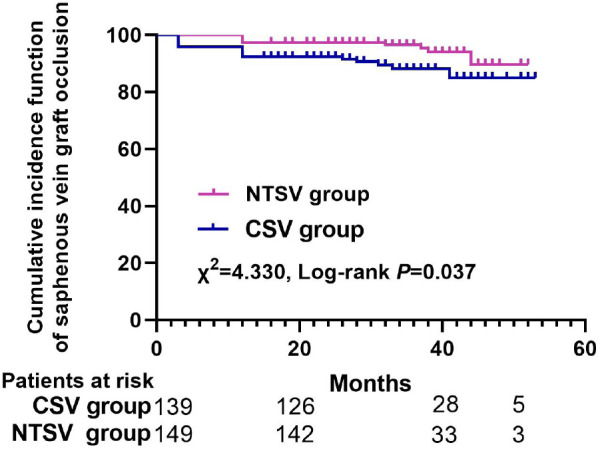
The cumulative incidence function of saphenous vein graft occlusion between the conventional saphenous vein (CSV) harvesting technique group and the no-touch saphenous vein (NTSV) harvesting technique group.

Patients who died or refused angiographic evaluation were excluded from follow-up; early postoperative (mean postoperative time 3.3±1.1 months) multidetector CT angiography (n=158) or coronary angiography (n=135) to evaluate the anastomotic sites and patency of the grafts were performed in 89.9% of study patients (293 of 326). At the first postoperative year (12.0±1.5 months), 100% of all patients (288 of 288) underwent graft evaluation using coronary angiography (n=155) or multidetector CT angiography (n=133) (Somatom Definition dual-source scanner; Siemens Medical Solutions, Forchheim, Germany). The three-month and one-year SVG patency rates were significantly higher in NTSV group than in CSV group (99.6% *vs.* 96.2%; *P*<0.001 and 97.3% *vs.* 93.1%; *P*<0.001), respectively. As shown in Figure 3, the two matched groups presented a significantly different cumulative survival freedom from SVG occlusion for the longer follow-up period in Kaplan-Meier curves (χ^^2^^=4.330, log-rank *P*=0.037). For stratified log-rank test, the two matched groups also developed a significantly different cumulative survival freedom from SVG occlusion (χ^^2^^=4.747, stratified log-rank *P*=0.029).

### Long-Term Outcomes

**Fig. 4:**
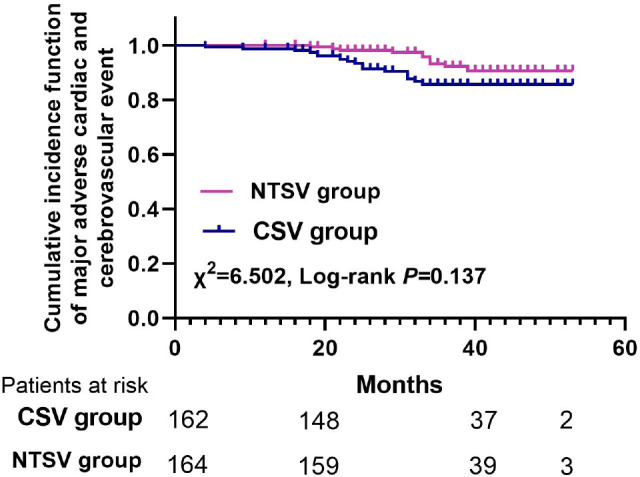
The cumulative incidence function of major adverse cardiac and cerebrovascular event between the conventional saphenous vein (CSV) harvesting technique group and the no-touch saphenous vein (NTSV) harvesting technique group.

There were 162 patients in the CSV group and 164 patients in the NTSV group who underwent a successful follow-up, which reached 97.8% of the cohort. There were five deaths during follow-up, with the duration of 36 to 54 months and median of 43 months. No significant difference between the two matched groups was found for all-cause mortality (1.6% CSV *vs.* 1.1% NTSV; *P*=0.652). The rates of persistent leg wound pain and surgical intervention were not significantly different (7.4% CSV *vs.* 8.4% NTSV; *P*=0.683 and 4.2% CSV *vs.* 4.6% NTSV; *P*=0.792). The rate of MACCE was not statistically significantly different between the two groups, but there was a tendency favoring the no-touch technique (9.6% CSV *vs.* 4.8% NTSV; *P*=0.067). As shown in Figure 4, the two matched groups received a similar cumulative incidence function of MACCE in Kaplan-Meier curves (χ^^2^^=6.502, log-rank *P*=0.137).

## DISCUSSION

The key finding of this study was that the NTSV grafts had statistically significantly higher patency rates at both the 3-month and 1-year angiographic follow-ups as compared with the CSV grafts.

The two matched groups received a significantly different cumulative survival freedom from SVG occlusion for the longer follow-up period in Kaplan-Meier curves (χ^^2^^=4.330, log-rank *P*=0.037). A longitudinal, prospective, randomized clinical trial was performed to determine the effect of the NTSV and reported a significantly higher patency rate of NTSV at 1.5, 8.5, and 16 years postoperatively^[^9^-^11^]^. Deb et al.^[^12^]^ performed a meta-analysis study that showed a marked reduction of vein graft occlusion. Also, a recent multicenter randomized clinical trial by Tian et al.^[^13^]^ from China, including 2,655 patients, demonstrated that the NTSV group had significantly higher proportion of graft patency compared with the CSV group at both three and 12 months. Our findings reinforce the conclusions of these studies.

In the present study, the use of SVG conduits was 72.6% in 508 patients who underwent OPCABG. It was shown that nearly 80% of patients used the SVG, which was consistent with other centers^[^14^-^16^]^. In the total cohort, there was no difference in demographic data and preoperative risk factors between the two groups, except for a higher proportion of diabetics (*P*=0.026) in the CSV group than in the NTSV group. However, after the PS matching, the difference in diabetics between the groups was no longer observed (18.3% [31 patients] NTSV *vs.* 20.1% [33 patients] CSV; *P*=0.781). Patients in the matched NTSV group compared with the matched CSV group were similar in characteristic and perioperative clinical data. In the perioperative data of the two groups, we found that the NTSV group had longer hospitalization time before surgery for total cohort (*P*=0.009) and propensity-matched cohort (*P*=0.013), probably because the patients in the NTSV group needed to be carefully preoperatively reviewed and evaluated, especially the Doppler ultrasonography mapping took more time when this technique was initially started at our center. In addition, the NTSV group had a longer duration of operation for total cohort (*P*=0.017) and propensity-matched cohort (*P*<0.001), which was mainly due to the unskilled work for the early period that this technique was initially brought in, suggesting that a learning curve of the surgical practice and the hard training was very important. In our study, there was no significant difference in surgical mortality between the two groups. Infection, renal insufficiency, re-exploration for bleeding, myocardial infarction associated with CABG, perioperative stroke, and prolonged ventilation were similar between the two groups. No significant difference between the two matched groups was found for all-cause mortality. The rate of MACCE was not statistically significant between the two matched groups but there was a tendency favoring the no-touch technique (9.6% CSV *vs.* 4.8% NTSV; *P*=0.067). The two matched groups received a similar cumulative incidence function of MACCE in Kaplan-Meier curves (*P*=0.137), which revealed the similar in-hospital and follow-up outcomes between the two groups. Our results were consistent with previous studies^[^10^-^13^,^17^,^18^]^.

Regarding leg wound complication, patients in the NTSV group developed a higher proportion of persistent exudation postoperatively (*P*=0.032) and skin numbness (*P*=0.001) than those in the CSV group. Our results were in agreement with the two important randomized trials mentioned before^[^12^,^13^]^. Nonetheless, the persistent pain or surgical intervention for the leg wound were similar between the two groups in the follow-up period, which indicated that the wound complications are mostly mild and less likely to affect long-term life quality. The use of skin bridges or drains as described by Kim et al.^[^18^]^ may lead to a reduction in the incidence of leg wound infections. Thus, careful incision closure intraoperatively and wound management postoperatively were necessary to reduce leg wound complications.

### Limitations

There are several limitations to our study. First, the study has the usual limitations of retrospective investigations, although all consecutive patients who met the inclusion criteria were enrolled and PS matching analysis was also performed to overcome these limitations. Furthermore, the follow-up period was relatively short. Besides, the competitive graft flow was not further analyzed in this study, which may affect the angiographic outcomes to some extent. Finally, our study focused on patients submitted to OPCABG, so whether different surgical techniques have the same impact on patency rates of NTSV grafts remains unknown.

## CONCLUSION

The NTSV harvesting technique provided an improvement of the patency rate after OPCABG as compared with the conventional vein harvesting approach. To reduce the incidence of leg wound complications it was crucial a learning curve of the surgical practice and dedicated training.

**Table:** 

Authors’ Roles & Responsibilities	
ZP	Drafting the work and revising it critically for important intellectual content; final approval of the version to be published
RZ	Drafting the work and revising it critically for important intellectual content; final approval of the version to be published
ZL	Substantial contributions to the analysis of data for the work; final approval of the version to be published
YL	Substantial contributions to the analysis of data for the work; final approval of the version to be published
YY	Drafting the work and revising it critically for important intellectual content; final approval of the version to be published
XY	Substantial contributions to the conception of the work; final approval of the version to be published
KH	Substantial contributions to the conception of the work; final approval of the version to be published
